# Analytical Study of Porous Organosilicate Glass Films Prepared from Mixtures of 1,3,5- and 1,3-Alkoxysilylbenzenes

**DOI:** 10.3390/ma14081881

**Published:** 2021-04-09

**Authors:** Md Rasadujjaman, Xuesong Wang, Yanrong Wang, Jing Zhang, Valeriy E. Arkhincheev, Mikhail R. Baklanov

**Affiliations:** 1 Department of Microelectronics, North China University of Technology, Beijing 100144, China; binzhouwxs@163.com (X.W.); zhangj@ncut.edu.cn (J.Z.); m_baklanov@hotmail.com (M.R.B.); 2 Department of Physics, Dhaka University of Engineering & Technology, Gazipur 1707, Bangladesh; 3 Laboratory of Applied Physics, Advanced Institute of Materials Science, Ton Duc Thang University, Ho Chi Minh City 729000, Vietnam; valeriy.arkhincheev@tdtu.edu.vn; 4 Faculty of Applied Sciences, Ton Duc Thang University, Ho Chi Minh City 729000, Vietnam; 5 Research and Education Center “Technological Center”, MIREA—Russian Technological University, 119454 Moscow, Russia

**Keywords:** organosilicate glass, low-*k* films, benzene bridges, pore structure, FTIR, ellipsometric porosimetry, Young’s modulus, dielectric constant, thermal stability

## Abstract

Organosilicate glass (OSG)-based porous low dielectric constant (low-*k*) films with different molar ratios of 1,3,5-tris(triethoxysilyl)benzene to 1,3-bis(triethoxysilyl)benzene bridging organic groups (1:3 and 1:7) were spin-on deposited, followed by a soft bake in air and N_2_ at 150 °C and hard bake in air and N_2_ at 400 °C. Non-ionic template (Brij^®^30) concentrations were varied from 0 to 41 wt% to control the porosity of the films. The chemical composition of the matrix of the films was evaluated and discussed with the shrinkage of the film during the curing, refractive indices, mechanical properties, *k*-values, porosity and pore structure. The chemical composition of the film cured in both air and *N*_2_-containing ambient were evaluated and compared. The benzene bridging groups containing films change their porosity (0 to 43%) but keep the pore size constant and equal to 0.81 nm when porosity is lower than 30%. The *k*-value decreases with increasing porosity, as expected. The films containing benzene bridge have higher a Young’s modulus than plasma-enhanced chemical vapor deposition (PECVD) methyl-terminated low-*k* films with the same porosity and show good hydrophobic properties after a hard bake and close to the values reported for 1,4-benzene-bridged films. The fabricated films show good stability after a long time of storage. However, the improvement of mechanical properties was lower than the values predicted by the published literature data. It was concluded that the concentration of 1,3,5-benzene bridges was below the stiffness threshold required for significant improvement of the mechanical properties. The films show UV-induced luminescence with a photon energy of 3.6 to 4.3 eV. The luminescence is related to the presence of oxygen-deficient-type defects or their combination with organic residues. The most intensive luminescence is observed in as-deposited and soft bake samples, then the intensity is reduced after a hard bake. It is assumed that the oxygen-deficient centers form because of the presence of Si–OC_2_H_5_ groups in the films and the concentration of these centers reduces when all these groups completely transformed into siloxane (Si–O–Si).

## 1. Introduction

Since the late 1990s, microelectronic technology has been replacing traditional SiO_2_ and Al in back-end-of-line (BEOL) technology with low dielectric (low-*k*) materials and low resistivity (Cu, Co, Ru, etc.) metals. The implementation of low-*k* materials and low-resistivity metals in BEOL is required to reduce the signal propagation delay (resistance-capacitance *RC*-delay) in interconnect structure, and also to reduce power dissipation and crosstalk noise between the metal lines [1,2]. However, low-*k* dielectrics are subjected to different mechanical and thermal stresses during the various integration processes of Cu/low-*k* structures, which, in turn, may lead to a fracture of the low-*k* dielectric and interfacial delamination due to the low mechanical strength [3].

Many different materials have been evaluated during the last 20 years as potential low-*k* candidates [4,5]. Currently, organosilicate glasses (OSG) have been selected as the most suitable for modern BEOL integration technology, although other candidates, such as metal organic frameworks, amorphous boron nitride and some others are still under study [4,6,7]. OSG low-*k* materials are also termed carbon-doped oxides (SiCOH) and have a structure similar to traditional silicon dioxide, where a part of oxygen-bridging atoms is replaced with terminal methyl groups. These methyl groups provide hydrophobic properties of OSG material and allow to avoid (or minimize) adsorption of water molecules that have extremely high dielectric constants (k ≈ 80) and therefore easily degrade the low *k*-value of selected dielectrics. However, the ≡Si–CH_3_ terminal groups also affect the mechanical properties by reducing the silicon network connectivity number in the low-*k* skeleton [8]. An additional problem with mechanical properties is related to the porosity of the films that reduces the stiffness of low-*k* materials [9,10,11,12]. Therefore, it is very important to ensure the preservation of good mechanical properties while decreasing the dielectric constant *k* by increasing the porosity [13,14].

The common way to improve the mechanical properties of OSG low-*k* films is to increase the network connectivity through the processing routes such as post-deposition curing to create more bridging bonds between the silicon atoms. However, the necessary level of hydrophobicity can be achieved if the concentration of terminal methyl groups is not lower than a certain critical value (about 10 to 12%). Recently, the introduction of organic bridging groups (methylene, ethylene, benzene, etc.) into the silica network (Si–O–Si) has become popular [14,15,16], since their introduction makes it possible to increase the silica network connectivity without a reduction of the total concentration of carbon-containing groups. The structure modification by bridging groups also reduces the density of OSG films and the bond polarizability (similar to terminal methyl groups) [17], which are beneficial for the improvement of dielectric properties. Furthermore, the incorporated bridging groups can form films with ordered porosity (periodic mesoporous organosilica, PMO) that would improve mechanical properties because of optimized structure and the higher bending rigidity of Si–C–Si bonds than that of Si–O–Si [5,15,18]. However, according to the recent experimental observations, the improvement of mechanical properties with the help of linear bridges (methylene, ethylene, benzene, etc.) was not so significant as expected from theoretical analysis, especially in the case of porous films [3,18,19,20].

Recently, Burg et. al. [21] proposed and designed the hyperconnected network architecture by using 1,3,5-silyl benzene precursors, where each silicon atom can be connected to the five other nearest silicon neighbors. Then, the 1,3,5-benzene bridging group structure connects each silicon atom to two others via carbon bridges that share one common Si–C bond while maintaining the ability of a silicon atom to connect with three others via Si–O–Si bonds. It has been reported [21,22,23] that the OSG hyperconnected hybrid network architectures, in which the connectivity of silicon atoms within the network goes beyond its chemical coordination number, creates a hyperconnected network that can dramatically improve the mechanical properties to the values higher than of fully dense silica while maintaining a low density [21].

The same research group has also shown that not only a hyperconnected network but also precursors geometry, carbon bridging length, bridging structural elements and the molecular geometry of organic carbon linkers are the factors that can control the overall structural stiffness of the organosilicate glasses. According to the results of the simulation, the 1,3,5-benzene network seems to be the most promising film whose highest bulk modulus could be about 50 GPa [23]. They also supposed the dielectric constant of OSG film without artificial porosity to be around 3 because of its reduced density.

However, the implementation of a hyperconnected network with carbon-containing groups still has challenges that need to be explored experimentally. The introduced aromatic groups increase the films dielectric constant in comparison with low-*k* films containing terminal methyl groups with the same porosity. Therefore, it might be a drawback of the improved mechanical properties. The introduced carbon-containing bridging groups do not provide sufficient hydrophobicity of the films and therefore an additional introduction of terminal groups might be needed. The thermal and ultraviolet (UV)-induced luminescence properties of carbon-bridged groups can also be an issue [24]. Finally, the hyperconnected structures lead to poor mechanical reliability due to their brittle nature, which is a significant challenge for their reliable integration into microelectronic devices [22].

Only one experimental work related to the fabrication and study of a low-*k* film with a hyperconnected structure has been reported by Liu et.al. [25]. The OSG films with different 1,3- and 1,3,5-benzene ratio were fabricated. The components ratio in the precursor solutions were characterized by nuclear magnetic resonance (NMR). The dielectric and mechanical properties were studied. The maximum Young’s modulus is 8.07 GPa with a reasonable *k* value (*k* = 2.99), and the best k value is 2.4 with a Young’s modulus of 4.47. However, how the 1,3,5- and 1,3-benzene bridges affect the dielectric constant and mechanical property are not clarified yet. So, further study to investigate the relation between the 1,3,5-benzene network and the properties of OSG film is necessary. However, it seems that the proper 1,3,5-benzene content for the best film quality has not been disclosed. The stability of the film should be studied for application in ultra large-scale integration (ULSI).

**Figure 1:**
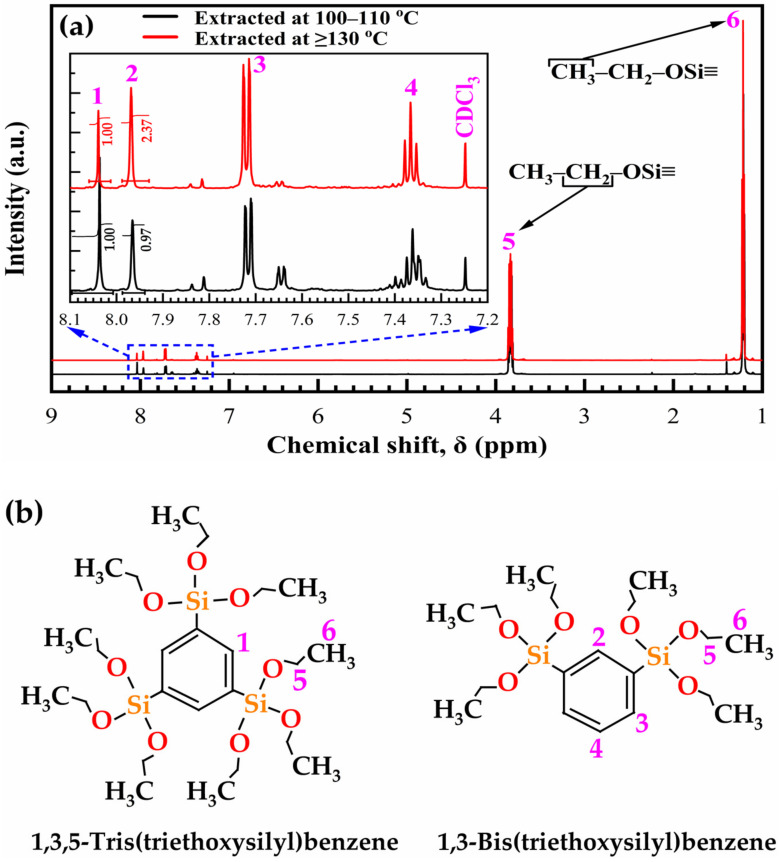
(**a**) ^1^H-NMR spectrum of two 135TTEB precursor solutions, one extracted at 100–110 °C and another one at ≥130 °C, where the signals are assigned as follows: 1 = δ 8.06 (s, 3H, ArH), 1,3,5-benzene (135TTEB), 2 = δ 7.97 (s, 1H, ArH), 1,3-benzene (13BTEB), 3 = δ 7.73 (m, 2H, ArH) 1,3-benzene (13BTEB), 4 = δ 7.38 (t, 1H, ArH), 1,3-benzene (13BTEB), 5 = δ 3.83 (q, J = 7.65 Hz, 18H, CH_2_), the methylene group of CH_3_–CH_2_–OSi, 6 = δ 1.22 (J = 7.71 Hz, 27H, CH_3_), the methyl group of CH_3_–CH_2_–OSi. The signal of the CDCl_3_ solvent appears at 7.24 ppm, and (**b**) chemical structure of 1,3,5-Tris(triethoxysilyl)benzene and 1,3-Bis(triethoxysilyl)benzene.

In this work, we present the synthesis of hybrid OSG materials with a narrow pore size distribution by using mixtures of 1,3,5- and 1,3-benzene-bridged alkoxysilyl precursors (Figure 1b). These precursors are used to understand the potential of the benzene-bridged precursors as supports for low-*k* materials. Two different molar ratios of these precursors (1:3 and 1:7, respectively) are used for the fabrication of the film. The goal of this work is to understand the effect of this ratio on different properties of OSG films when they are deposited together with a porogen. The advantages of benzene bridges for the dense films have been demonstrated theoretically, but there is a need to apply porous OSG films for advanced microelectronics practice, in which case, the effect of these bridges on the low-*k* matrix is less obvious. The pore structure, dielectric properties, mechanical properties, hydrophobicity and thermal stability of the films were studied under different porogen content and annealing conditions. The presented results are committed to the verification and optimization of novel hyperconnected molecular network architectures for improving mechanical properties, which is important for BEOL integration technology.

## 2. Materials and Methods

### 2.1. Materials

1,3,5-Tris(triethoxysilyl)benzene (135TTEB) precursor solution with two different molar ratios of 1,3,5-benzene (135TTEB) and 1,3-benzene (13BTEB) bridging groups were prepared by using commercially available precursors and published a literature procedure [21,25]. 1,3,5-Tribromobenzene (TBB, 98%, Sigma-Aldrich LLC., Shanghai, China), Iodine (I, 99%, Sigma-Aldrich LLC., Shanghai, China), Tetraethylorthosilicate (TEOS, 99%, Energy-Chemical Shanghai Co., Ltd., Shanghai, China), Tetrahydrofuran (THF, 99.5%, Energy-Chemical Shanghai Co., Ltd., Shanghai, China), n-hexane (99%, Energy-Chemical Shanghai Co., Ltd., Shanghai, China), and magnesium (99.5%, Zhejiang Lianshuo Biological Technology Co. Ltd., Zhejiang, China) were used to synthesis 135TTEB precursor solution. The strategy employed to prepare the benzene bridge porous OSG films used evaporation-induced self-assembly (EISA) with a non-ionic Brij^®^30 (C_12_H_25_(OCH_2_OCH_2_)_4_OH, molar mass 362 g/mol, template (porogen) (Sigma-Aldrich, LLC., Shanghai, China), 1-methoxy-2-propanol (1m2p, 99.5%, Sigma-Aldrich LLC., Shanghai, China) and HNO_3_ (65 to 70%, Alfa Aesar, Thermo Fisher Scientific, Shanghai, China) to control the porosity of the film during the spin-on deposition. The synthesis of the 135TTEB precursor solution and the films deposition procedure have been described in detail in our previous publication [25]. To prepare porous films, 135TTEB precursor solution was mixed with porogen taking into account different weight ratios of the template to the precursor—0, 17, 23, 29, 33, 38, and 41%, respectively. Two different molar ratios (one distillation at 100–110 °C and the other distillation at ≥130 °C) of 135TTEB and 13BTEB bridging groups solution, confirmed by NMR spectra, were prepared for spin-on coating. Benzene bridge precursor in 1-methoxy-2-propanol (solvent) was mixed with different Brij^®^30 porogen with an appropriate amount of aqueous solution of HNO_3_ (acid catalyst) to prepare a spin-on coating solution. The final solution was collected and kept for 24 h at room temperature in a dark closed bottle. Silicon substrates were cleaned by diluted HF aqueous solution and dried by the flow of pure nitrogen before the preparation of the film. Then, the solutions were spin-coated on 100 mm Si wafers at 2500 rpm for 50 s. The resulting films were “soft baked” (SB) at 150 °C for 30 min in both air and N_2_. Finally, the films were “hard baked” (HB) at 400 °C, in the air for 30 min and N_2_ for 60 min to increase the condensation degree of the film precursors and to remove the residual porogen.

### 2.2. Characterization

The molecular structure of as-prepared precursor solutions was confirmed by nuclear magnetic resonance (NMR) spectroscopy. NMR spectra were recorded with a JNM-ECZ600R spectrometer (JEOL, Beijing, China) operating at 400 MHz for 1H. The hydrogen nuclear spin precesses in a 94 Tesla magnetic field to generate an NMR signal of about 400 MHz. The chemical composition of the series of deposited films was measured by using FT/IR-6300 Fourier-transform infrared (FTIR) spectrometer (JASCO, Shanghai, China) in transmission mode with a resolution of 4 cm^−1^ (at least 64 scans) in the range 4000 to 400 cm^−1^. A pure silicon wafer cut from the same wafer was used for the film deposition and the background spectra for FTIR measurement were obtained using the same wafer. To analyze the chemical composition, all the obtained spectra are normalized to the highest Si–O–Si peak.

The thickness and index of refraction (RI), porosity, and pore size distribution of the films were measured by using ellipsometric porosimetry (EP). The system uses a SENpro spectroscopic ellipsometer (SENTECH, Berlin, Germany) with λ = 350–850 nm. The angle of light incidence was fixed at 70°. Heptane vapor diluted by N_2_ carrier gas was used as an adsorptive. The films’ open porosities were calculated as the volume of condensed liquid adsorptive (*V*) from RI values measured during the heptane adsorption by using the Lorentz–Lorenz equation [26]. The calculation of the pore size of the mesoporous film is based on the Kelvin equation [27]. The size of the micropores is calculated by using the Dubinin–Radushkevich equation adapted for EP [26,27]. EP was also used to estimate the Young’s modulus of porous low-*k* films and it calculates Young’s modulus simultaneously with the measurement of pore size distribution in a fast and easy way [28].

Capacitance-voltage (CV) was measured using wafer probing (Cascade Microtech Summit 12000, Santa Clara, CA, USA) and a precision Agilent B1500A inductance, capacitance and resistance meter (Santa Clara, CA, USA) to obtain the dielectric constant. For this purpose, the metal-insulator-semiconductor (MIS) planar capacitors were used. The top electrodes were formed by sputtering 120-nm-thick aluminum through a metal shadow mask on the benzene bridge organosilicate films/silicon structure. It should be noted that a 120-nm-thick continuous aluminum coating is also deposited on the backside of the silicon wafer. The resistivity of silicon wafers was 1 to 10 µOhm-cm for these measurements. The capacitance was measured in different frequencies from 1 kHz to 1 MHz at room temperature and it was stable until 100 kHz per unit area. To characterize the surface hydrophilicity of benzene bridge films, the water contact angle (WCA) was measured on an INNUO CA100A (Shanghai JZ Instrument and Equipment, Shanghai, China).

The luminescence data of the benzene bridge organosilicate films was measured by FP-8300 Photoluminescence (PL) spectrometer (JASCO, Shanghai, China) by using Xe arc lamp (150 W). The excitation energy of photon was used from 6.2 eV and the emission spectra range were measured in the wavelength from 210 to 750 nm.

## 3. Results and Discussion

### 3.1. Molecular Structure of 135TTEB-Bridged Precursor

The 135TTEB precursor solution was extracted at two different temperatures—one precursor solution (i) at 100–110 °C and another one (ii) at ≥130 °C. The molecular structures of the two 135TTEB precursor solutions were confirmed by NMR testing after dissolution in deuterated chloroform (CDCl_3_). The ^1^H-NMR (600 MHz, chloroform-d) spectra of as-synthesized precursor samples are shown in Figure 1a and the chemical structure of 1,3,5-Tris(triethoxysilyl)benzene and 1,3-Bis(triethoxysilyl)benzene are shown in Figure 1b. It has been proven that the destruction of precursor during the synthesis happened certainly for both materials when Si–C bonds were broken and formed ethyl silicate and 1,3-benzene structural units. All the peaks are already reported in the literature [25,29]. The various observed chemical shifts are summarized in Table 1.

The height of the integral curve in the NMR spectrum represents the number of hydrogen nuclei that cause the formant. The molar ratio was calculated from the first peak of 135TTEB (at δ = 8.06) and the second peak of 13BTEB (at δ = 7.97). The detailed molar ratio calculations can be found in the Supplementary Materials (SI). The molar ratio of 135TTEB and 13BTEB is about 0.73:2.05 (denoted as 1:3) extracted at 100 to 110 °C and is about 0.39:2.77 (denoted as 1:7) extracted at ≥130 °C in the precursor solution. The ^1^H NMR spectra confirm that the 135TTEB precursor was prepared successfully.

### 3.2. Influence of 135TTEB and 13BTEB Bridge Group Ratio and Curing Ambient on the Properties of OSG Low-k Films

Table 2 shows the comparative data for benzene-bridged OSG materials with different 135TTEB and 13BTEB ratios and different porogen concentrations. The data presented in Table 2 gives the properties of the films versus 1,3,5- and 1,3-benzene bridging group concentrations and curing ambient.

### 3.3. Change of Thickness and Refractive Index on Curing in Air and N_2_ for Different Molar Ratio (1:3 and 1:7) Benzene-Bridged OSG Films

**Figure 2:**
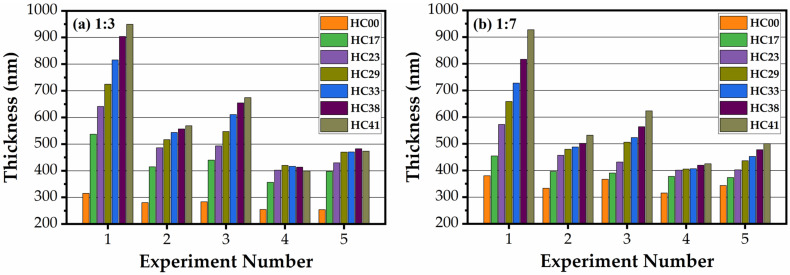
Dependence of film thickness on the template (0 to 41 wt%, numbered from HC00 to HC41) and benzene-bridge content for (**a**) 1:3, and (**b**) 1:7 materials. The experiment number corresponds to (1) as-deposited (AD), (2) after soft baked in air for 30 min (SB-Air), (3) soft baked in N_2_ for 30 min (SB-N_2_), (4) hard baked in air for 30 min (HB-Air), and (5) hard baked in N_2_ for 60 min (HB-N_2_).

**Figure 3:**
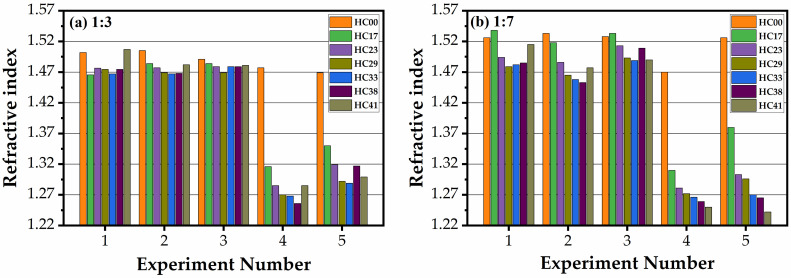
Change of refractive index of films (0 to 41 wt%, numbered from HC00 to HC41) and benzene-bridge content for (**a**) 1:3, and (**b**) 1:7 materials. The experiment number corresponds to (1) as-deposited (AD), (2) after soft baked in air for 30 min (SB-Air), (3) soft baked in N_2_ for 30 min (SB-N_2_), (4) hard baked in air for 30 min (HB-Air), and (5) hard baked in N_2_ for 60 min (HB-N_2_).

Figure 2 and Figure 3 show the change in thickness and refractive index happening during the curing of all the studied samples in the air and N_2_ (1:3 benzene bridge (Figure 2a and Figure 3a) and 1:7 benzene bridge (Figure 2b and Figure 3b)). The experiment numbers correspond to (1) as-deposited (AD), (2) after soft baked in air for 30 min (SB-Air), (3) soft baked in N_2_ for 30 min (SB-N_2_), (4) hard baked in air for 30 min (HB-Air), and (5) hard baked in N_2_ for 60 min (HB-N_2_). After coating, the films became thicker with the increase of porogen concentration from 0 to 41 wt% (experiment number 1). All the AD films contain a certain amount of solvent (1-methoxy-2-propanol) and template. It is seen that the thickness of the films reduces after soft baked at 150 °C both in air and N_2_. It is clearly seen that the thickness reduces more for air-cured films than N_2_-cured ones and the trend is similar for both 1:3 (Figure 2a) and 1:7 (Figure 2b) benzene bridge ratio materials. The reason is more efficient oxidation and the removal of template residues that covered the pore wall surface and embedded into the film matrix [30]. Their removal leads to shrinkage of the film matrix and reduces the thickness of the film. The films deposited without template (HC00) have the smallest thickness after deposition and the shrinkage during the consecutive steps of curing is not significant (for instance, it changes from 315.3 nm to 254.9 nm for air-cured 1:3 material, see Table 1). The change of thickness during the complete cycles of curing is much higher for the samples with porogen (from 949.4 nm to 398.9 nm for air-cured 1:3 material, see Table 1). Therefore, the content of the porogen with the solvent exceeded the amount of matrix material twice.

The change of the indices of refraction of all films were measured simultaneously, with the thickness measurements (Figure 3). The refractive index (RI) of the samples deposited without porogen slightly decreases during the curing (the samples presented in Figure 3b, experiment number 5, is an exception). It is interesting that the RI of templated samples has not changed after a soft baked (experiment number 2 and 3) while the thickness reduction was significant. It suggests that the solvent (1-methoxy-2-propanol and water) evaporation mainly happened during this step, and almost all template remained inside the pores. As mentioned above, air curing is more efficient than nitrogen curing because of oxidation of the template residue embedded into the matrix, but we already do not see much difference in RI values after experiments 3 and 4 because the curing time was sufficient for complete porogen removal. The difference between the samples deposited with different template concentration reflects the difference in the full porosity of these films.

### 3.4. Chemical Composition of (135TTEB:13BTEB = 1:3 and 1:7) Benzene-Bridged OSG Films

To investigate the effect of chemical composition changes, a series of films were deposited on low-doped Si wafers with 135TTEB:13BTEB (1:3 and 1:7) benzene bridge ratios and a different Brij30 surfactant concentration from 0 to 41 wt%. The changes in the chemical composition of the films were obtained with varying surfactant content by FTIR for air and N_2_ cured films.

**Figure 4:**
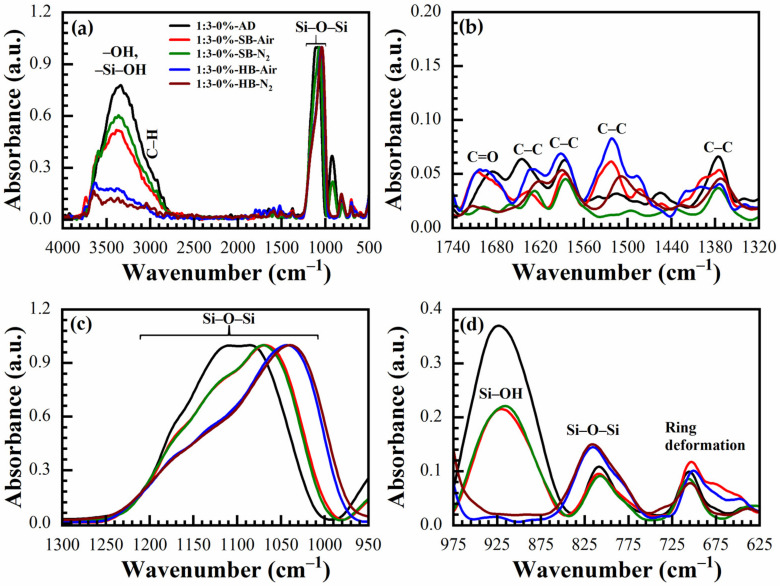
FTIR spectra of benzene-bridged (135TTEB:13BTEB = 1:3 material) organosilicate films without porogen loading, where (**a**) full FTIR spectra; and (**b**–**d**) are the parts of the full FTIR spectra. The films are as-deposited (AD), soft baked (SB) at 150 °C in the air (SB-Air) and N_2_ (SB-N_2_) for 30 min, hard baked (HB) at 400 °C in the air for 30 min (HB-Air) and in N_2_ (HB-N_2_) for 60 min.

**Figure 5:**
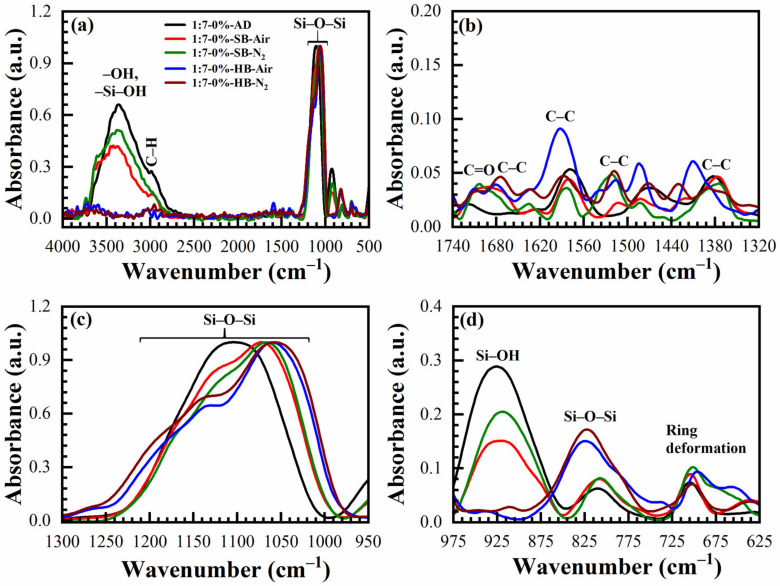
FTIR spectra of benzene-bridged (135TTEB:13BTEB = 1:7 material) organosilicate films without porogen loading, where (**a**) full FTIR spectra; and (**b**–**d**) are the parts of the full FTIR spectra. The films are as-deposited (AD), soft baked (SB) at 150 °C in air (SB-Air) and N_2_ (SB-N_2_) for 30 min, hard baked (HB) at 400 °C in air for 30 min (HB-Air) and in N_2_ (HB-N_2_) for 60 min.

**Figure 6:**
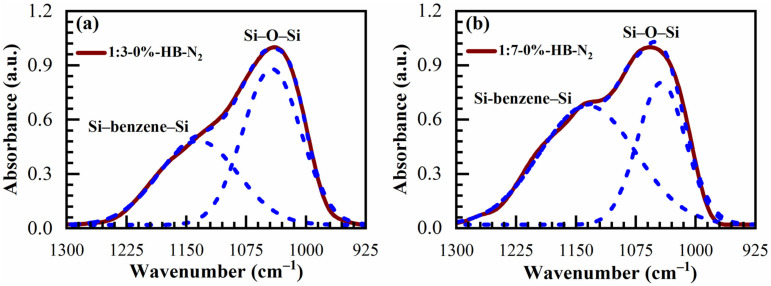
Deconvoluted FTIR spectra in the range of 1300–925 cm^−1^ for the completely cured (**a**) 1:3, and (**b**) 1:7 materials cured in N_2_ for 60 min (HB-N_2_). It can be seen that the spectra are deconvoluted into two peaks related to Si–O–Si vibration at 1043 cm^−1^ and also from the bond of Si with benzene ring at 1135 cm^−1^ because of the presence of benzene bridges in our films.

The IR absorbance spectra of AD, SB at 150 °C in the air (SB-Air) and N_2_ (SB-N_2_), HB at 400 °C in the air for 30 min (HB-Air) and N_2_ for 60 min (HB-N_2_) with benzene-bridge ratio OSG films are shown in Figure 4 and Figure 5. The films were obtained from the different 135TTEB:13BTEB ratio materials (1:3 (Figure 4) and 1:7 (Figure 5)) without the use of a template do not show significant differences. The most intensive peaks in FTIR spectra are located at the wavenumber (WN) range 1200–1000 cm^−1^. The broad peak 1200–1050 cm^−1^ of the as-deposited sample (before curing) most probably corresponds to ≡Si–OC_2_H_5_ groups present in the precursors (Figure 1). The curing leads to (i) hydrolysis reactions ≡Si–O–C_2_H_5_ + H_2_O → ≡Si–OH + C_2_H_5_–OH and (ii) condensation reactions ≡Si–OH + HO–Si≡ → ≡Si–O–Si≡ + H_2_O and the peak of ≡Si–OC_2_H_5_ transforms to silica-like bonds ≡Si–O–Si≡ matrix peak at 1050–1000 cm^−1^. The hydrolysis reaction partially happens during the sol fabrication and storage, and this is the reason why AD film already contains Si–OH groups. The complete condensation of Si–OH groups happens only during the HB, and these are the reasons why AD and SB films still show the presence of Si–OH groups and adsorbed water as peaks at ~920 cm^−1^ and also broad absorption at 3000–3700 cm^−1^ (Figure 4d). It is clear (Figure 4c) that complete condensation of ≡Si–OH groups is not happening during the soft bake and only a hard bake is needed to complete the reaction. It is necessary to mention that the peak in the range 1300–925 cm^−1^ in the completely cured samples still can be deconvoluted into two peaks at 1135 cm^−1^ and 1043 cm^−1^ related to Si–O–Si vibration and also from the bond of Si with a benzene ring [31] because of the presence of benzene bridges in our films (Figure 6a,b).

The siloxane ≡Si–O–Si≡ symmetric stretching vibration is located near 800 cm^−1^ [32]. The intensity of this peak increases after HB and this fact supports our conclusion that more ≡Si–O–Si≡ groups are formed after condensation of ≡Si–OH groups. The film deposited without porogen does not contain any C–H groups (3000–2800 cm^−1^). The C–C stretching vibration originating from the presence of benzene rings are only visible in the region between 1740–1320 cm^−1^ (Figure 4b and Figure 5b). The peaks associated with the benzene C–C vibration are not so pronounced due to their small absorption coefficient in comparison with Si–O bonds. The 1,3,5-trisubstituted C–C stretching vibration can be occurring at 1600 and 1500 cm^−1^, and the 1,3-disubstituted C–C stretching vibration can be occurring at 1610 and 1590 cm^−1^ [33]. In our films, the characteristic bands at 1590 cm^−1^ are correlated to the 1,3-disubstituted benzene C–C stretching vibration. In addition, the AD 1:3 material contains more surface silanol groups and adsorbed water than 1:7 material. It might suggest that the 1,3-benzene-bridged OSG material is more hydrophobic than 1,3,5-benzene-bridged one. The most reasonable explanation is that benzene groups protect (shield) some silanol groups because of their relatively large size. This complicates the condensation reactions of silanol groups located near the benzene ring. It is also easy to imagine that the shielding effect of 1,3,5-benzene groups is higher than that of 1,3-benzene, due to the more branched structure (see Figure 1). Consequently, more OH groups remain, which are adsorption sites for the water molecules. The surface silanols are the centers of water molecule adsorption (wide band at 3750–3000 cm^−1^) and as it was mentioned above, they can degrade the low-*k* properties. The weak C–H stretching vibration of ring hydrogen is also observed at ~3040 cm^−1^. There are certain peaks at ~1700 cm^−1^ which may be related to the C=O saturated stretching vibration [33]. They appear after HB and can reflect partial oxidation of the deposited films. However, the peak intensity is always very small.

**Figure 7:**
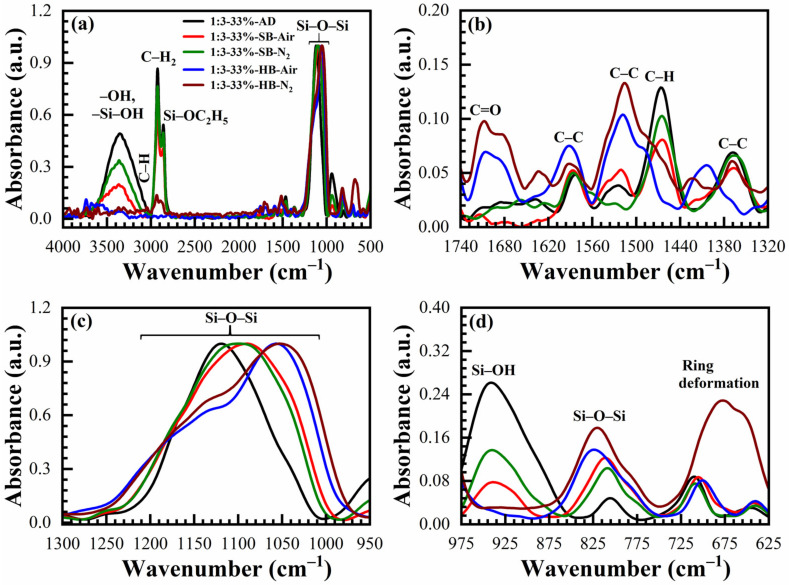
FTIR spectra of benzene-bridged (135TTEB:13BTEB = 1:3 material) organosilicate films with 33 wt% porogen loading, where (**a**) is the full FTIR spectra and (**b**–**d**) are the parts of the full FTIR spectra. The films are as-deposited (AD), soft baked (SB) at 150 °C in the air (SB-Air) and N_2_ (SB-N_2_) for 30 min, hard baked (HB) at 400 °C in the air for 30 min (HB-Air) and in N_2_ for 60 min (HB-N_2_).

**Figure 8:**
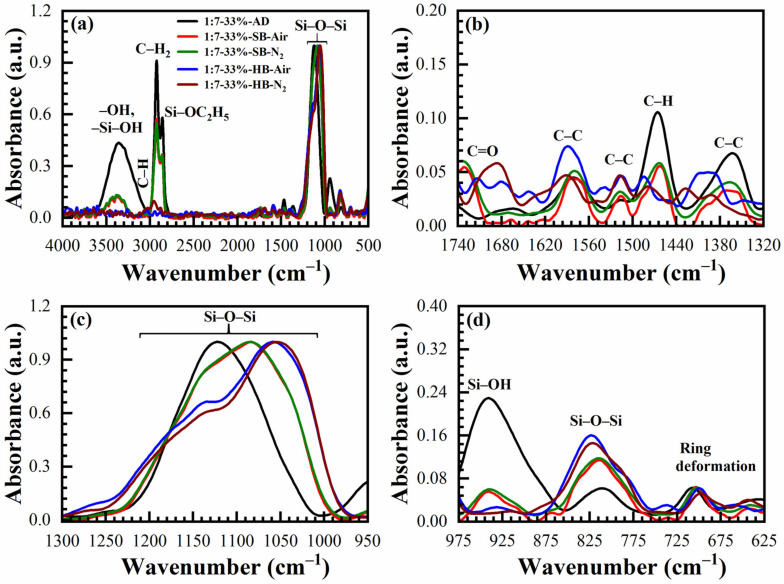
FTIR spectra of benzene-bridged (135TTEB:13BTEB = 1:7 material) organosilicate films with 33 wt% porogen loading; where (**a**) is the full FTIR spectra and (**b**–**d**) are the parts of the full FTIR spectra. The films are as-deposited (AD), soft baked (SB) at 150 °C in the air (SB-Air) and N_2_ (SB-N_2_) for 30 min, hard baked (HB) at 400 °C in the air for 30 min (HB-Air) and in N_2_ for 60 min (HB-N_2_).

The chemical compositions of OSG films deposited with 17 to 41 wt% porogen loading cured in the air and N_2_ are also deduced from FTIR spectra for comparison. The typical FTIR spectra of a sample deposited with 33 wt% porogen loading films are shown in Figure 7 (1:3 material) and Figure 8 (1:7 material). Transformation of the peaks located in the range 1040–1130 cm^−1^ is similar to the films deposited without template (Figure 4 and Figure 5) and a similar interpretation can be applied based on the transformation of ≡Si–OC_2_H_5_ groups to ≡Si–O–Si≡ groups as a result of the condensation reaction. The only difference is that the films contain a high concentration of CH_x_ groups that originated from the template and are visible in the range 2800–3000 cm^−1^ together with a peak related to ≡Si–OC_2_H_5_ (2845 cm^−1^) (Figure 7a and Figure 8a) and also near 1460 cm^−1^. The curing process (both SB and HB) gradually increases the concentration of siloxane bridges (≡Si–O–Si≡ groups at 1050 cm^−1^ and 800 cm^−1^), reduces the concentration of CH_x_ bonds (2800–3000 cm^−1^ and 1460 cm^−1^) as well as the concentration of ≡Si–OC_2_H_5_ bonds (2845 cm^−1^). The template (porogen) removal occurs by thermal decomposition of the template and desorption of its volatile fragments. Decomposition of porogen can also be initiated by UV light, plasma, atomic hydrogen and oxygen [1]. The peaks related to adsorbed moisture (3000–3700 cm^−1^) and the silanol group (~950 cm^−1^) also reduces gradually.

The concentration of the C–H_x_ and ≡Si–OH groups remaining in SB films depends on porogen concentrations. Both of these groups are more efficiently removed from the films after HB in the air and N_2_. It is interesting to note that the films after HB are similar to the spectra presented in Figure 4 and Figure 5, without porogen loading. The changes in film composition are also visible at 1275–975 cm^−1^ for both materials (Figure 7 and Figure 8) due to the introduction of the porogen.

**Figure 9:**
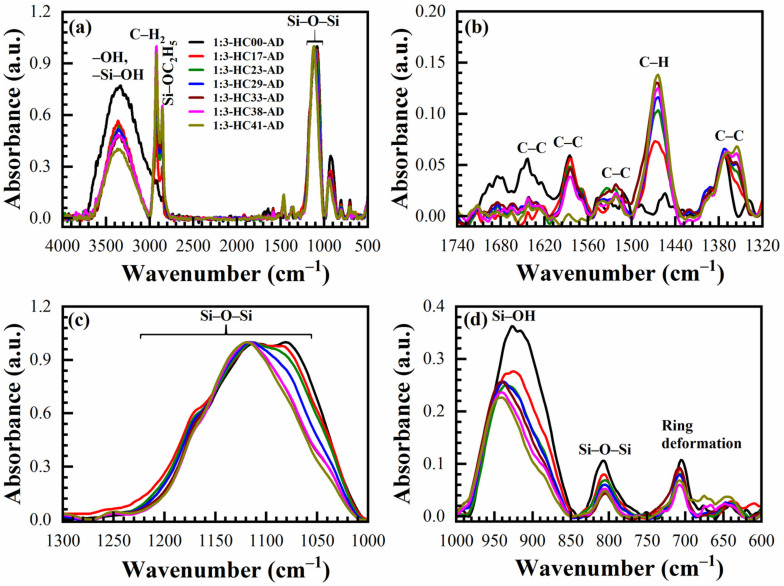
FTIR spectra of 1:3 ratio material as-deposited (AD) films with different template loadings (0 to 41 wt%, films numbered from HC17 to HC41) where (**a**) is the full FTIR spectra and (**b**–**d**) are the parts of the full FTIR spectra.

Figure 9 shows the FTIR spectra of as-deposited low-*k* films with a 1:3 ratio material deposited with different porogen concentration (0 to 41 wt%). The concentration of CH_x_ bonds (2925 cm^−1^ and 1460 cm^−1^) increases with porogen loading, as expected. The highest amount of adsorbed water is observed in the film deposited without the template, then this amount slightly decreases with the template concentration. It is interesting that the films with a low template concentration are more ≡Si–O–Si≡ rich. The possible explanation is that ≡Si–OC_2_H_5_ groups have a lower probability of a cross-linking condensation reaction because of a steric factor related to the presence of the template. Quite similar phenomena have been observed in the 1:7 material but their detailed analysis can be found in Supplementary Materials (Figure S1).

**Figure 10:**
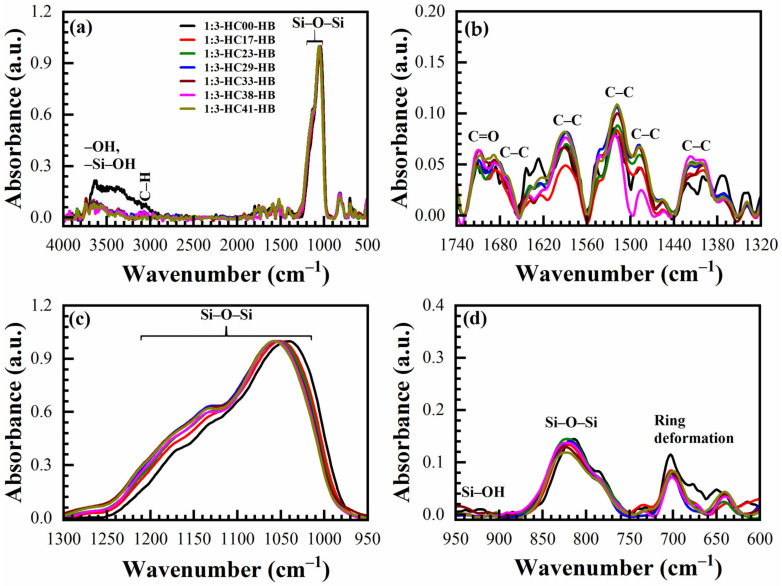
FTIR spectra of 1:3 ratio material films hard baked (HB) in the air (30 min) with different template loadings (0 to 41 wt%, films numbered from HC17 to HC41) where (**a**) is the full FTIR spectra and (**b**–**d**) are the parts of the full FTIR spectra.

The consecutive SB (150 °C, 30 min) and HB (400 °C, 30 min in the air) removes ≡Si–OC_2_H_5_, CH_x_ bonds and the FTIR spectra of all films deposited with different template concentration are becoming similar (Figure 10). The almost complete removal of ≡Si–OC_2_H_5_ and CH_x_ bonds suggests that this regime of curing was quite efficient for porogen removal. In the samples cured in N_2_, we need a curing time that is twice as long to achieve the same efficiency of curing (see Supplementary Materials Figure S2).

### 3.5. Porosity and Pore Size Distribution (Ellipsometric Porosimetry Data)

**Figure 11:**
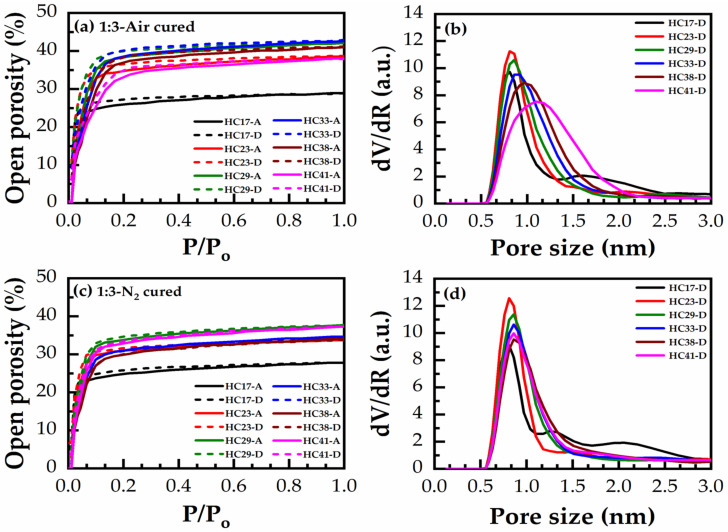
Heptane adsorption (A)/desorption (D) isotherms (heptane relative pressure, P/P_o_ (ratio of current pressure P to saturated vapor pressure P_o_ at room temperature) vs. open porosity) of 1:3 material (**a**) hard baked in the air for 30 min (air-cured) (**c**) hard baked in N_2_ for 60 min (N_2_-cured), and the corresponding pore size distribution (PSD) in (**b**,**d**), respectively, calculated from desorption (D) isotherms, showing the effect of varying the porogen concentration from 17 to 41 wt% (films numbered from HC17 to HC41).

**Figure 12:**
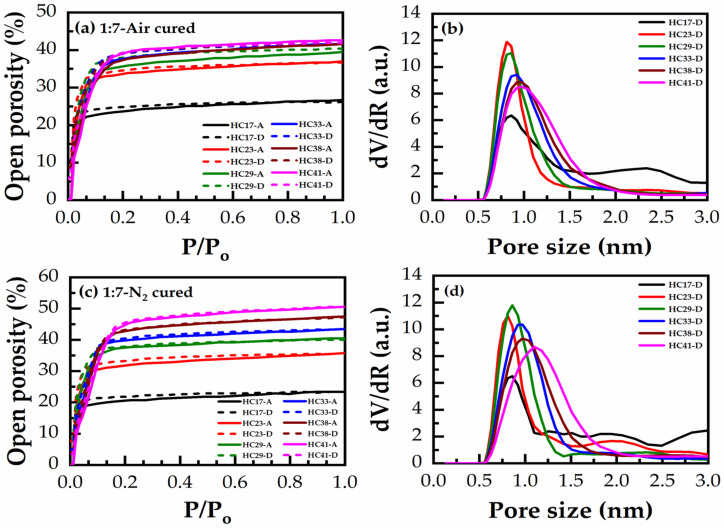
Heptane adsorption (A)/desorption (D) isotherms (heptane relative pressure, P/P_o_ vs open porosity) of 1:7 material (**a**) hard baked in the air for 30 min (air-cured) (**c**) hard baked in N_2_ for 60 min (N_2_-cured), and the corresponding pore size distribution (PSD) in (**b**,**d**), respectively, calculated for desorption (D) isotherms, showing the effect of varying the porogen concentration from 17 to 41 wt% (films numbered from HC17 to HC41).

Adsorption/desorption isotherms of heptane vapors and the calculated pore radius distribution (PRD) for 1:3 and 1:7 materials with various porogen content from 17 to 41 wt% are shown in Figure 11 and Figure 12. In these isotherms: (a,b) are HB in the air for 30 min and (c,d) are HB in N_2_ for 60 min. The general tendency of porosity change is quite similar for all films.

**Figure 13:**
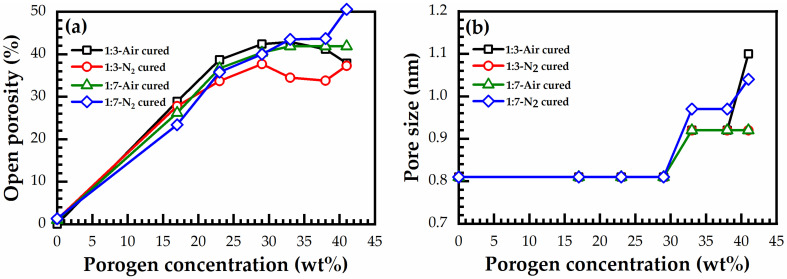
(**a**) Open porosity and (**b**) pore size versus porogen concentration for 1:3 and 1:7 materials, after being cured at 400 °C in the air for 30 min (air-cured), and in N_2_ for 60 min (N_2_-cured).

Figure 13 demonstrates the effects of bridging groups on the pore size and porosity after different curing environments. First, the measured porosity increases proportionally to the porogen concentration, then it reaches saturation, starting from a porogen concentration of 30 wt% (Figure 13a). The maximum porosity was reached in N_2_ cured 1:7 material (50% at 41 wt% porogen loading). However, the average value of the maximum porosity after saturation was about 43%. The smallest and most constant pore size of 0.81 nm is observed until 30 wt% porogen loading (Figure 13b). A further increase of porogen does not change porosity but it increases the pore size.

The presented EP data shows that an unusual property of benzene-bridged samples in comparison with alkylene-bridged and methyl-terminated materials [34,35] is their very small pore size. The pore radius value is constant and equal to 0.81 nm until a porogen concentration lower than 30% (Figure 13b). The adsorption–desorption isotherms do not have a hysteresis loop (Figure 12a), which suggests the quasi-cylindrical shape of micropores without internal voids [34,35]. The small pore size can make them more attractive for the integration in ULSI interconnects because of better compatibility with ultrathin barrier layers [36]. However, the controllable increase of porosity to values higher than 30% was problematic. The maximum porosity was reached at about 40% but the pore size at porosity > 30% starts to increase. The possible reason is that some pores at porosity > 30% begin to collapse, increasing the size of adjacent pores and making it difficult to further increase porosity.

### 3.6. Young’s Modulus and Dielectric Constant

**Figure 14:**
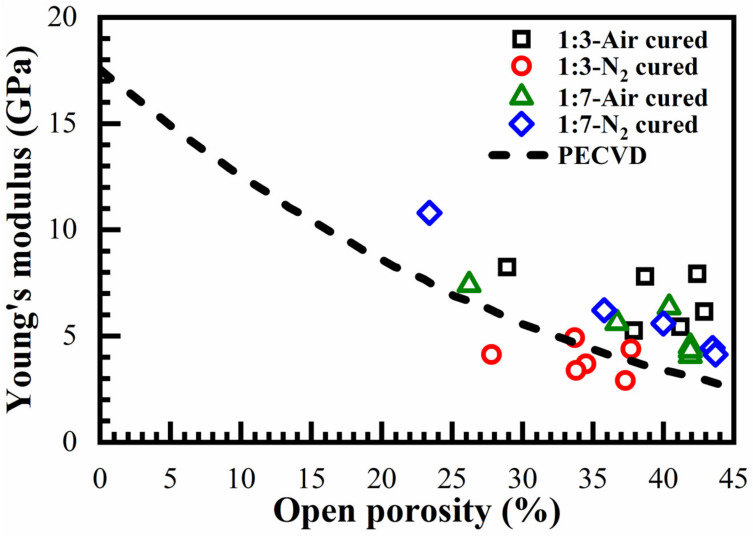
Dependence of Young’s modulus on porosity and benzene bridge ratios (1:3 and 1:7) for films cured in the air (air-cured) and in N_2_ (N_2_-cured). The short dash line corresponds to the change of Young’s modulus calculated for plasma enhanced-chemical vapor deposition (PECVD) materials.

Figure 14 shows Young’s modulus values measured by ellipsometric porosimetry (EP). The presented data is also compared with Young’s modulus values obtained for plasma-enhanced chemical vapor deposition (PECVD) methyl-terminated OSG films (dashed curve) and reported in the paper [37]. One can see that all the deposited films except N_2_-cured 1:3 have Young’s modulus values higher than in PECVD films. The observation of a relatively low Young’s modulus of N_2_-cured 1:3 film can be explained by RI data presented in Figure 2. The relatively high RI values obtained for the N_2_-cured 1:3 film suggests that the curing process was not complete.

It should be noted that the measured Young’s moduli were close to the values reported for the low-*k* samples with 1,4 benzene bridge [38] and significantly lower than the values predicted for 1,3,5-benzene-bridged hyperconnected materials reported in the work [21]. Usually, the stiffness of materials is related to the topological connectivity of the system as a whole. In two types of our films, the concentration of 1,3-bridged molecules was three and seven times higher than the 1,3,5-bridged molecules. Therefore, a reasonable explanation of the measured Young’s moduli values can be based on a model that considers our films as a composition based on a random distribution of 1,3,5-benzene-bridged clusters in 1,3-benzene-bridged media. In a two-component system with very different stiffness characteristics, the Young’s modulus must suddenly increase from a less rigid material to a harder material when the concentration of rigid material exceeds a certain critical concentration, called the percolation threshold [39]. In the three-dimensional case, the percolation threshold is close to 0.3 [39]. Although one of our films has a composition close to this value, unfortunately, in this work we did not achieve the expected increase in Young’s modulus. The reason for this difference is as follows: For the electrical current flow (which is more often described by the percolation threshold), it is sufficient to have at least one conductive path. Meanwhile, to ensure good rigidity important for mechanical properties, it is necessary to use a network of links (a branched cluster of several links). In other words, the conduction is provided by the network of wire channels, and the strength is provided by the more branched and intertwined structure of the percolation paths. Such a difference in the structure, apparently, leads to different values of the percolation thresholds for conductivity and the threshold for hardness. Good examples comparing stiffness and electrical percolation thresholds have been demonstrated in the references [39,40]. Snarskii et al. [40] showed that in the frameworks of effective medium approximation, the electrical percolation thresholds and the stiffness threshold have different values in the three-dimensional case: percolation threshold = 0.3 and stiffness threshold = 0.5. As another example, we can refer to work [39], in which randomly distributed networks of carbon nanotubes (CNTs) were studied using theoretical analysis as well as numerical modelling. It was shown that the average number of crossings on each CNT is the only dominant factor for both electrical percolation thresholds and stiffness thresholds, but for electrical percolation thresholds, it is about 3.7 and 5.2 for the hardness threshold. Consequently, the concentration of 1,3,5-benzene groups (30%) assumed as sufficient for the percolation threshold were not sufficient for the stiffness threshold. A more detailed study of these issues is planned in our future work.

**Figure 15:**
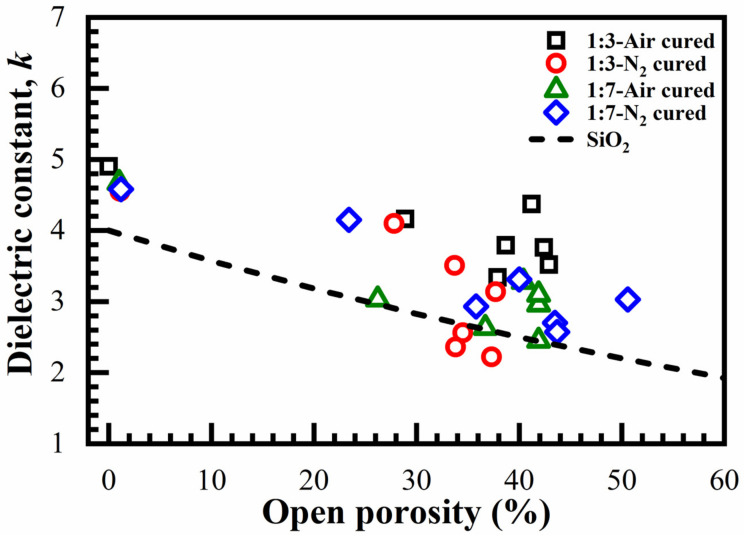
Dependence of the dielectric constant, *k* (at 100 kHz) on porosity and concentration of benzene bridges for films bake in the air and N_2_ for 1:3 and 1:7 materials. The SiO_2_ reference is calculated according to the Clausius–Mossotti equation assuming the *k* of dense SiO_2_ is equal to 3.9.

The dielectric properties of the films with 1:3 and 1:7 ratio materials are shown in Figure 15. The *k*-value was extracted from the C–V measurements as described in the experimental part. The *k* values calculated for the films cured in the air and those cured in N_2_ have no consistent differences. However, the air-cured 1:3 samples have a higher *k*-value. The dashed curve shows the Clausius–Mossotti curve for porous silica. Furthermore, it shows that the *k*-values are higher for a higher concentration of 1,3,5-benzene groups (1:3).

### 3.7. Hydrophobicity and Hydrophilicity of the Films Surface

**Figure 16:**
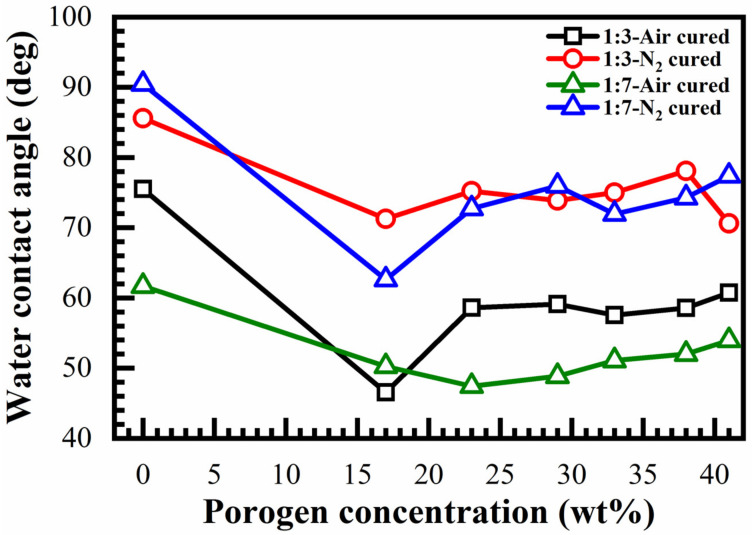
Influence of porogen loading on the water contact angle (WCA) of benzene bridge OSG films hard baked in the air (air-cured) and N_2_ (N_2_-cured) for 1:3 and 1:7 benzene bridge ratio materials.

The influence of the film composition, curing environment and porogen concentration on the water contact angle (WCA) of benzene-bridged OSG films are presented in Figure 16. The film deposited without porogen has the highest contact angle (hydrophobic). It is difficult to see the difference between 1:3 and 1:7 materials, probably because the curing environment has a much higher effect. The samples cured in N_2_ clearly have a higher contact angle. The reason for this difference is the effect of the remaining template residue after curing in an N_2_ environment. It has already been reported that the remaining template residue renders the low-*k* surface more hydrophobic and protects against the impact of oxidizing chemistries [30].

### 3.8. Effect of Storage

**Figure 17:**
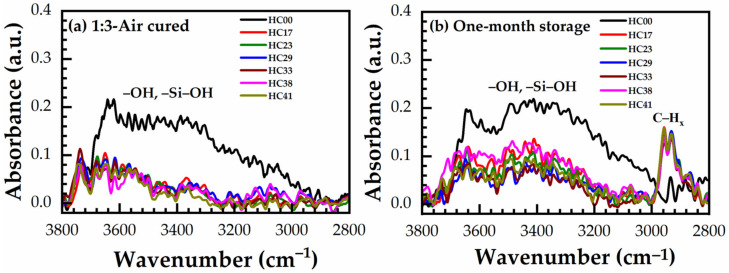
Fingerprint region of FTIR spectra of (**a**) air cured for 30 min (just cured) and the corresponding (**b**) one-month stored samples with different porogen (0 to 41 wt%, films numbered from HC17 to HC41) loadings.

The low-*k* films are microporous and actively adsorb (accumulate hydrocarbon residues) moisture from the atmosphere and it increases their *k*-value. To know the effect of storage of the benzene-bridged OSG films, time stability has also been studied for air-cured and N_2_-cured 1:3 material. Figure 17a,b shows the fingerprint region (3800–2800 cm^−1^) of FTIR spectra of hard baked in the air for 30 min (just cured) and the corresponding one-month stored 1:3 material. Figure S3a,b also shows the FTIR spectra of a film hard baked in N_2_ for 60 min (just cured) and the corresponding one-month stored 1:3 material (see Supplementary Materials). As shown in Figure 17b and Figure S3b, some amount of organic residues (3000–2800 cm^−1^) and moisture (3800–3000 cm^−1^) have been accumulated after one month of storage in the laboratory. The adsorbed organic residue and moisture can be removed relatively easily by annealing at 300 °C for 10 min. Therefore, all these changes are fully reversible, and no irreversible degradation of low-*k* material is observed during the storage.

It should be noted that the sample HC00 deposited without porogen practically does not accumulate organic residues, as this film is not porous and has a small surface area compared with the samples deposited with the porogen. This statement also supports that the amount of adsorbed CH_x_ species increases with the increase of the porogen amount and is especially pronounced in the 1:3 material (Figure S3b).

**Figure 18:**
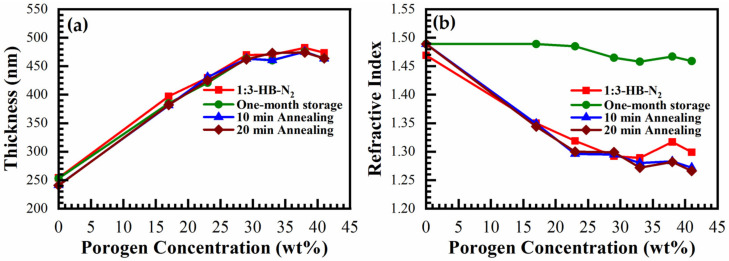
Change of (**a**) thickness and (**b**) refractive index during the storage. It shows that initial thickness and refractive index restored after 10 min annealing at 300 °C in air.

Figure 18 shows the change of thickness (*d*) (Figure 18a) and refractive index (RI) (Figure 18b) of N_2_-cured (just cured) films (1:3 material) after one-month storage in the air and after thermal annealing for 10 min and 20 min at 300 °C. It can be seen that the storage of the film in the air increases the RI due to the accumulation of organic residues and moisture. However, the adsorbed residues can be completely removed by thermal annealing, and the original *d* and RI are restored after 10 min of annealing in air at 300 °C. The film thickness does not change during storage and thermal annealing. The storage of air-cured films also shows the same tendencies (data not shown).

### 3.9. Thermal Stability

**Figure 19:**
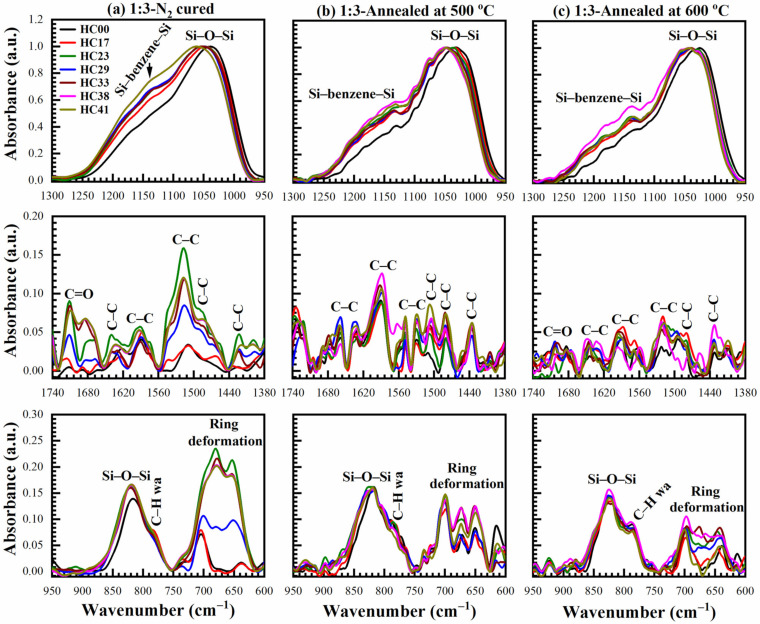
Ranges of FTIR spectra for 1:3 material after (**a**) hard baked (HB) at 400 °C in N_2_ for 60 min (N_2_-cured), (**b**) annealing at 500 °C for 30 min, and (**c**) annealing at 600 °C for 30 min with different porogen (0 to 41 wt%, films numbered from HC17 to HC41) loadings.

To get an idea about thermal stability, the samples deposited with a benzene-bridge ratio of 1:3 and 1:7 cured for 60 min in N_2_ (HB-N_2_) were additionally annealed in N_2_ at 500 °C and 600 °C. Then, the chemical compositions of the annealed samples were analyzed by FTIR. The results presented in Figure 19 (1:3 material) demonstrate the comparison of three fingerprint regions of FTIR spectra (a) 1:3-N_2_ cured (just cured), (b) annealed at 500 °C for 30 min, and (c) annealed at 600 °C for 30 min. There are no significant changes in chemical composition from just N_2_-cured films to annealing in N_2_ at 600 °C films. The deformation in the fingerprint region 1300–950 cm^−1^ is related to cage deformation, which is related to the Si–O–Si angle due to residue removal. Moreover, the accumulated C–H_x_ groups (3000–2800 cm^−1^) during the storage are removed during the annealing, as already shown in Figure 18. The peaks at ~775 cm^−1^ are related to C–H wagging. FTIR spectra of 1:3 N_2_-cured samples in the range 750–625 cm^−1^ may contain ring deformation [33,41]. The thermal stability analysis of N_2_-cured 1:7 material can be found in the Supplementary Materials (Figure S3).

**Figure 20:**
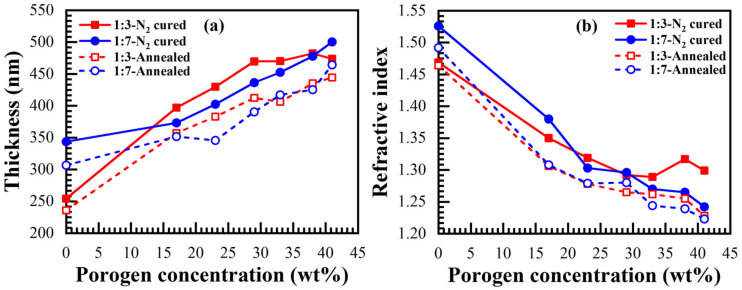
Effect of annealing at 600 °C for 30 min (annealed) on (**a**) thickness and (**b**) refractive index of 1:3- and 1:7-benzene bridge ratio materials N_2_-cured at 400 °C for 60 min (just N_2_-cured) with different porogen concentrations.

Minor changes of thickness and refractive index are observed after additional annealing of the just-cured 1:3 and 1:7 materials (Figure 20).

**Figure 21:**
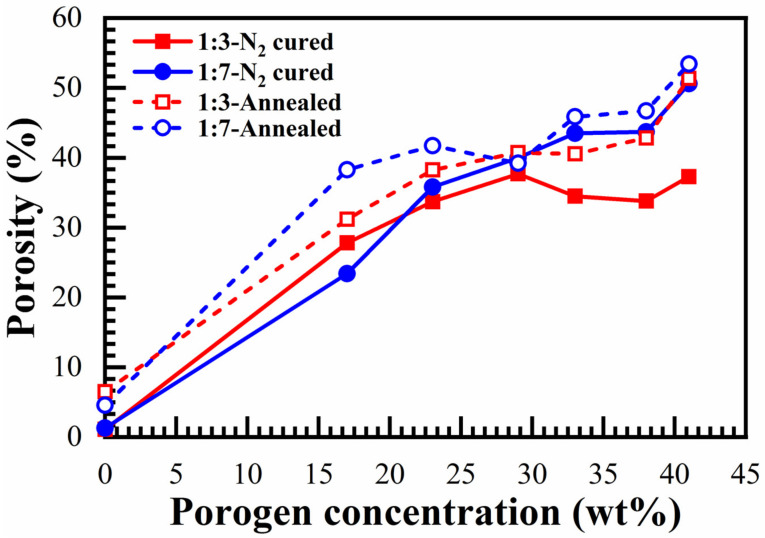
Effect of annealing at 600 °C for 30 min (annealed) on the porosity of 1:3- and 1:7-benzene bridge ratio N_2_-cured at 400 °C for 60 min (N_2_-cured) samples with different porogen (0 to 41 wt%) loadings. The porosity of both N_2_ annealing samples is calculated by using the Lorentz–Lorenz equation.

The porosities of the annealed films are also calculated with the Lorentz–Lorenz equation [26] by using the refractive index measured after annealing. Figure 21 shows the changes of porosity for different porogen content films after annealing the just-cured samples at 600 °C for 30 min.

### 3.10. UV-Induced Photoluminescence (PL)

**Figure 22:**
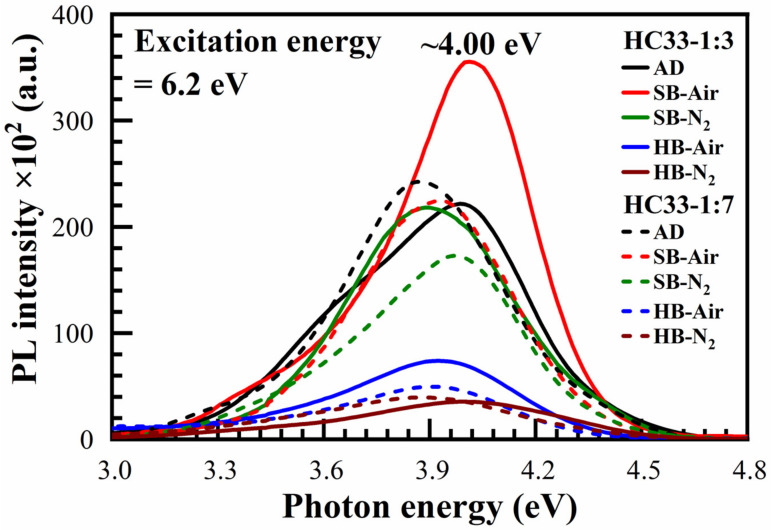
UV-induced PL spectra of 33 wt% porogen content films (numbered as HC33) for 1:3 and 1:7 benzene bridge ratio materials as-deposited (AD), soft baked (SB) at 150 °C for 30 min both in air (SB-Air) and N_2_ (SB-N_2_), hard bake (HB) at 400 °C for 30 min air (HB-Air) and 60 min in N_2_ (HB-N_2_) under 6.2 eV excitation.

UV-induced luminescence is known as a powerful tool for better understanding the nature of internal defects in dielectric films and their correlation with dielectric properties [42,43]. In particular, the results of studying luminescence in combination with other methods makes it possible to identify the types of oxygen vacancies that play an important role in the conductivity mechanisms. The room-temperature UV-induced PL spectra of 33 wt% porogen loading films (numbered as HC33) for both 1:3 and 1:7 materials as-deposited (AD), soft baked both in the air (SB-Air) and N_2_ (SB-N_2_), and hard baked both in the air (HB-Air) and N_2_ (HB-N_2_) films, are presented in Figure 22.

The observed emission at the energy range of 2.7–4.9 eV has already been found on SiO_2_ films and explained by the electronic transitions onto other oxygen-deficient-type defects or their combination with the organics [42,43]. Similar photoluminescences with separated peaks at 2.9, 3.3 and 4.3 eV have been observed in ethylene-bridged OSG films [44,45] and methyl-terminated OSG films [46]. Using the electron energy loss spectra, photoluminescence and data from the simulation within the density functional theory for the model SiCOH low-*k* structure confirms the presence of oxygen vacancy and divacancy in the studied films. We think that the photoluminescence spectra of benzene-bridged films also indicate the formation of oxygen-deficient centers similar to ethylene-bridged and methyl-terminated OSG films.

It is interesting that these defects exist in our AD films, then had a maximum intensity in SB 1:3 films while the emission intensity of 1:7 films reduced after SB. On the other hand, HB significantly reduced the luminescence intensity. These facts allow us to assume that oxygen-deficient centers form during the hydrolysis of ≡Si–OC_2_H_3_ groups. The hydrolysis reaction is almost completed during the hard bake and the concentration of these defects reduces.

## 4. Conclusions

1,3,5-Tris(triethoxysilyl)benzene (135TTEB) precursor solutions with two different molar ratios of 1,3,5-benzene (135TTEB) and 1,3-benzene (13BTEB) bridging groups were prepared by using 1,3,5-Tribromobenzene (TBB) and Tetraethylorthosilicate (TEOS). These precursors were then used for the spin-on deposition of low-*k* films with two different molar ratios of 1,3,5- and 1,3-benzene bridges equal to 1:3 and 1:7. Non-ionic templates (Brij^®^30) with concentrations varying from 0 to 41 wt% were introduced into precursor solutions to control the porosity of the deposited films. The deposited films were then soft baked in air and N_2_ at 150 °C and hard baked in air and N_2_ at 400 °C. The film compositions were analyzed using FTIR. It was shown that as-deposited and soft baked films are very hydrophilic due to the presence of silanol (SiOH) groups formed during hydrolysis of Si–OC_2_H_5_ groups, which occurs during aging of the precursor solution and soft baking of the films. Si–OH groups form Si–O–Si bonds as a result of a condensation reaction during the hard bake. Films deposited with porogen are also hydrophilic before curing, then become sufficiently hydrophobic after hard baking. The films fabricated with N_2_ cure show better hydrophobic properties.

A change of the template concentration from 0 to 41% allowed to change the film porosity from 0 to 30%. The pore size remained constant and equal to 0.81 nm. When the template concentration is higher than 30, porosity does not increase, but the pore size increases. This suggests that the pores tend to agglomerate at a high porogen concentration. Constant pore size is unusual for spin-on deposited low-*k* films where the pore size tends to increase with increasing porosity. As expected, the *k*-value decreases with increasing porosity. Films containing benzene bridges have a higher modulus of elasticity than methyl-terminated OSG low-*k* films deposited by using plasma-enhanced chemical vapor deposition (PECVD). The fabricated films show good stability after a long time of storage. However, the improvement in mechanical properties was lower than the values predicted in the literature for benzene-bridged OSG films. It was concluded that the concentration of 1,3,5-benzene bridges was below the stiffness threshold required for significant improvement of mechanical properties.

The films exhibit UV-induced luminescence with a photon energy of 3.6–4.3 eV. Luminescence is associated with the presence of oxygen-deficient-type defects as previously shown in OSG films with ethylene-bridged and methyl-terminated films. The most intense luminescence is observed in as-deposited and soft baked samples; intensity decreases after hard baking. It is assumed that the oxygen-deficient centers are formed during hydrolysis of Si–OC_2_H_5_ groups in the films. The concentration of these centers decreases when all these groups are completely converted into siloxane (Si–O–Si).

No significant differences were observed between films with different ratios of 1,3,5- and 1,3-benzene bridges, although their concentration in the precursor differed by more than two times (1:3 and 1:7). A qualitative explanation is that the expected improvement in Young’s modulus has a kind of percolation character and a real improvement of Young’s modulus needs an even higher concentration of 1,3,5-benzene bridging groups. Their concentration must exceed the stiffness threshold, which should be higher than the used concentration of the 1,3,5-benzene bridging groups. More extended experimental work and theoretical study are planned in our future work.

## Figures and Tables

**Table 1:** ^1^H-NMR (600 MHz, chloroform-d) chemical shift of two 135TTEB precursor solutions; one extracted at 100 to 110 °C and another one at ≥130 °C, where deuterated chloroform (CDCl_3_) was used as the solvent.

Chemical Shift, δ (ppm)	Peak Assignment
8.06	1,3,5-benzene (135TTEB)
7.97	1,3-benzene (13BTEB)
7.72	1,3-benzene (13BTEB)
7.38	1,3-benzene (13BTEB)
7.24	CDCl_3_
3.83	Methylene group of CH_3_–CH_2_–OSi
1.22	Methyl group of CH_3_–CH_2_–OSi

**Table 2:** Characteristics of porous OSG films with two different benzene bridge group ratios (135TTEB:13BTEB = 1:3 and 1:7) and different porogen concentrations (0 to 41 wt%, sample numbered from HC00 to HC41). The films were as-deposited (AD), soft baked (SB) at 150 °C for 30 min in both air and N_2_; and hard baked (HB) for 30 min in air and 60 min in N_2_. The two molar ratios of materials are leveled as 1:3-Air and 1:7-Air (air-cured) and 1:3-N_2_ & 1:7-N_2_ (N_2_-cured).

Sample Number	Status	As-Deposited (AD)		Soft Baked (SB) at 150 °C		Hard baked (HB) at 400 °C								
						*d* (nm)	RI	Porosity (%)		Pore Radius (nm)		WCA	YM from EP	*k*-Value
		*d* (nm)	RI	*d* (nm)	RI			Ads	Des	Ads	Des			
HC00	1:3-Air	315.3	1.501	280.5	1.505	254.9	1.477	-	-	-	-	75.52	-	4.90
	1:3-N_2_	311.2	1.503	283.8	1.491	254.2	1.469	-	-	-	-	85.59	-	4.55
	1:7-Air	366.6	1.526	333.4	1.533	315.7	1.470	-	-	-	-	61.68	9.98	6.46
	1:7-N_2_	427.9	1.520	379.9	1.528	343.7	1.526	-	-	-	-	90.46	11.05	5.59
HC17	1:3-Air	537.2	1.453	415.0	1.484	356.4	1.316	28.9	28.9	0.81	0.81	46.54	8.232	4.16
	1:3-N_2_	538.2	1.478	439.9	1.484	397.1	1.350	27.8	27.8	0.81	0.81	71.26	4.129	4.10
	1:7-Air	454.1	1.538	396.3	1.518	377.7	1.324	26.2	26.2	0.81	0.81	50.25	7.41	3.03
	1:7-N_2_	459.7	1.533	390.4	1.557	373.2	1.330	23.4	23.4	0.81	0.81	62.62	10.80	4.15
HC23	1:3-Air	641.0	1.475	486.2	1.477	402.5	1.285	38.5	38.7	0.92	0.81	58.60	7.795	3.79
	1:3-N_2_	643	1.478	493.1	1.479	429.7	1.319	33.7	33.7	0.81	0.81	75.22	4.919	3.51
	1:7-Air	572.4	1.494	456.8	1.486	401.9	1.281	36.7	36.7	0.81	0.81	47.43	5.62	2.62
	1:7-N_2_	541.4	1.512	431.6	1.513	402.4	1.303	35.8	35.8	0.81	0.81	72.73	6.21	2.93
HC29	1:3-Air	724.6	1.477	516.6	1.469	420.1	1.270	42.1	42.4	0.92	0.81	59.10	7.921	3.76
	1:3-N_2_	718.6	1.472	547.2	1.469	469.8	1.292	37.7	37.7	0.86	0.81	73.87	4.129	3.14
	1:7-Air	658.6	1.479	479.5	1.465	405.4	1.272	40.4	40.4	0.92	0.81	48.84	6.35	3.29
	1:7-N_2_	657.6	1.489	506.0	1.493	436.4	1.296	40.0	40.0	0.86	0.81	75.96	5.59	3.31
HC33	1:3-Air	815.4	1.464	543.6	1.467	416.3	1.268	42.6	42.9	0.92	0.92	57.53	6.142	3.52
	1:3-N_2_	823.2	1.470	610.8	1.479	470.2	1.289	34.5	34.5	0.86	0.92	74.99	3.631	2.56
	1:7-Air	727.7	1.482	488.0	1.458	401.5	1.266	41.9	41.9	0.92	0.92	51.09	4.51	2.96
	1:7-N_2_	737.1	1.482	523.5	1.489	452.5	1.270	43.5	43.5	0.97	0.97	71.96	4.43	2.7
HC38	1:3-Air	903.9	1.474	557	1.468	413.3	1.256	41.0	41.2	0.92	0.92	58.57	5.432	4.37
	1:3-N_2_	912.4	1.475	654.7	1.479	482.3	1.317	33.8	33.8	0.92	0.92	87.99	3.026	2.36
	1:7-Air	816.8	1.485	501.5	1.453	419.3	1.259	41.9	41.9	0.92	0.92	52.00	4.07	3.11
	1:7-N_2_	848.4	1.48	563.8	1.509	477.9	1.265	43.7	43.7	0.97	0.97	74.27	4.13	2.57
HC41	1:3-Air	949.4	1.510	568.6	1.482	398.9	1.285	38.0	37.9	1.25	1.10	60.74	5.248	3.34
	1:3-N_2_	948.1	1.504	674.3	1.481	473.7	1.299	37.3	37.3	0.86	0.92	70.62	2.899	2.22
	1:7-Air	927.7	1.515	531.9	1.477	425.0	1.250	41.9	41.9	0.92	0.92	53.96	4.31	2.45
	1:7-N_2_	918.3	1.521	623.4	1.49	500.3	1.242	50.6	50.6	1.04	1.04	77.38	2.95	3.03

*d* = thickness, RI = Refractive index, Ads = Adsorption, Des = Desorption, WCA = Water Contact Angle, YM = Young’s Modulus, *k* = dielectric constant.

## Data Availability

Data sharing is not applicable to this article.

## Supplementary Materials

The following are available online at https://www.mdpi.com/article/10.3390/ma14081881/s1, S1: Calculation of Molar Ratio from Mixtures of Two Precursors from NMR Spectra; Figure S1: FTIR spectra of 1:7 ratio as-deposited (AD) films with different template loadings (0 to 41 wt%, films numbered from HC17 to HC41); where (a) full FTIR spectra; and (b–d) are the parts of the full FTIR spectra, Figure S2: FTIR spectra of 1:3 ratio material films hard baked (HB) in N_2_ (60 min) with different template loadings (0 to 41 wt%, films numbered from HC17 to HC41); where (a) full FTIR spectra; and (b–d) are the parts of the full FTIR spectra, Figure S3: Fingerprint region (3800–2800 cm^−1^) of FTIR spectra of (a) N_2_ cured for 60 min (just cured) and the corresponding (b) one-month stored samples with different porogen (0 to 41 wt%, films numbered from HC17 to HC41) loadings. Figure S4: Ranges of FTIR spectra for 1:7 material after (a) hard baking (HB) at 400 °C in N_2_ for 60 min (N_2_ cured), (b) annealing at 500 °C for 30 min, and (c) annealing at 600 °C for 30 min with different porogen (from 0 to 41 wt%, films numbered from HC17 to HC41) loadings.
