# Supplementary material for: Analytical Study of Porous Organosilicate Glass Films Prepared from Mixtures of 1,3,5- and 1,3-Alkoxysilylbenzenes
Source: Materials (Basel). 2021 Apr 9;14(8):1881. doi: 10.3390/ma14081881 (PMC8069764; doi:10.3390/ma14081881)
Supplement: Supplementary file 1 [file materials-14-01881-s001.zip › Supplementary Materials corrected.pdf]

materials

Supplementary Material

Analytical Study of Porous Organosilicate Glass Films Prepared from Mixtures of 1,3,5- and 1,3-Alkoxysilylbenzenes

Md Rasadujjaman [1,2,*], Xuesong Wang [1], Yanrong Wang [1,*], Jing Zhang [1], Valeriy E. Arkhincheev [3,4] and Mikhail R. Baklanov [1,5]

[1] Department of Microelectronics, North China University of Technology, Beijing 100144, China; binzhouwxs@163.com (X.W.); zhangj@ncut.edu.cn (J.Z.); m_baklanov@hotmail.com (M.R.B.)
[2] Department of Physics, Dhaka University of Engineering & Technology, Gazipur, Gazipur 1707, Bangladesh
[3] Laboratory of Applied Physics, Advanced Institute of Materials Science, Ton Duc Thang University, Ho Chi Minh City 729000, Vietnam; valeriy.arkhincheev@tdtu.edu.vn
[4] Faculty of Applied Sciences, Ton Duc Thang University, Ho Chi Minh City 729000, Vietnam
[5] Research and Education Center "Technological Center", MIREA—Russian Technological University, 119454 Moscow, Russia
* Correspondence: rasadphy@duet.ac.bd (M.R.); wangyanrong@ncut.edu.cn (Y.W.)

S1. Calculation of Molar Ratio from Mixtures of Two Precursors from NMR Spectra

To calculate the molar ratio from mixtures of 1,3,5- and 1,3-benzene bridge precursors from NMR spectra we have used relative concentration determination method. For relative concentration determination, we were compared the integrals of interest with one another. For NMR test, we have confirmed the straight baseline, symmetrical sharp peak shape, straight integral curve at the place where there is no signal. It was set the maximum number of scans to get the signal to noise ratio around 255:1 with keeping the integration error <1%. However, the error in calculation of concentration can be slightly higher because of possible non-linearity of relative intensity of proton signal in NMR.

The molar ratio $M_{1,3,5\text{-benzene}}/M_{1,3\text{-benzene}}$ between two compounds 1,3,5-benzene and 1,3-benzene was determined by using the formula:

$$\frac{M_{1,3,5-benzene}}{M_{1,3-benzene}} = \frac{I_{1,3,5-benzene}}{I_{1,3-benzene}} \cdot \frac{N_{1,3-benzene}}{N_{1,3,5-benzene}}$$

where, $I_{1,3,5\text{-benzene}}$ and $I_{1,3\text{-benzene}}$ are the integral of 1,3,5-benzene and 1,3,5-benzene peaks respectively; $N_{1,3,5\text{-benzene}}$ and $N_{1,3\text{-benzene}}$ are the number of nuclei giving rise to the corresponding 1,3,5-benzene and 1,3,5-benzene peaks signal. The computer in an NMR instrument integrate the signals of interest, normalize for the number of protons, and perform a simple ratio analysis.

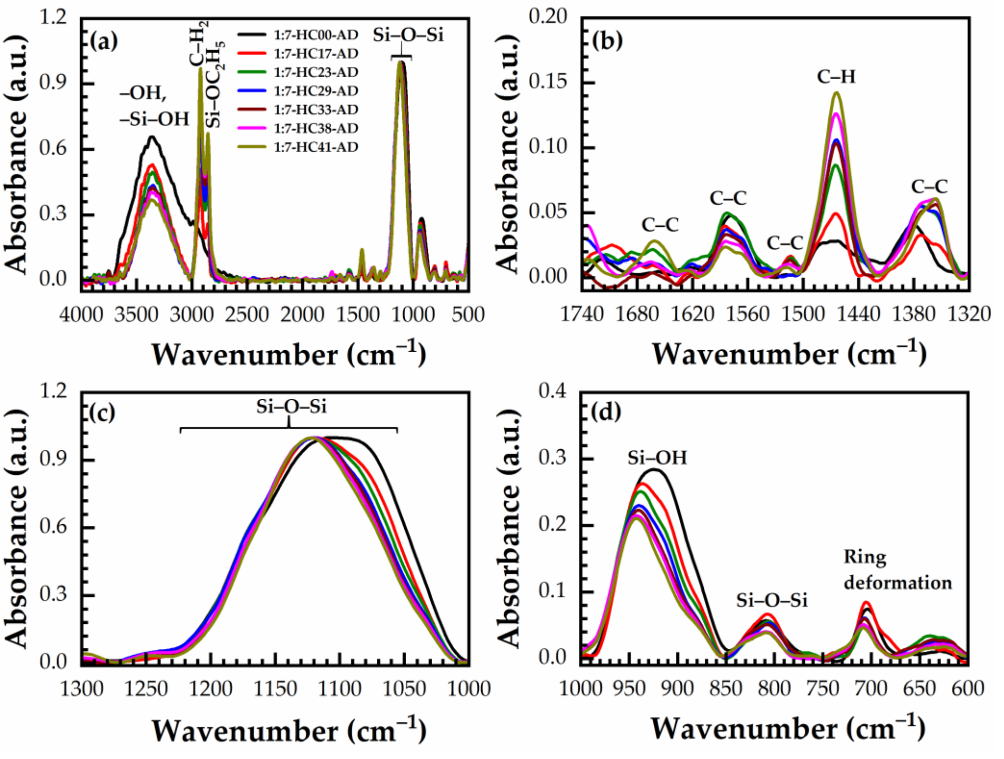

Figure S1. FTIR spectra of 1:7 ratio as-deposited (AD) films with different template loadings (0 to 41 wt%, films numbered from HC17 to HC41); where (**a**) full FTIR spectra; and (**b–d**) are the parts of the full FTIR spectra.

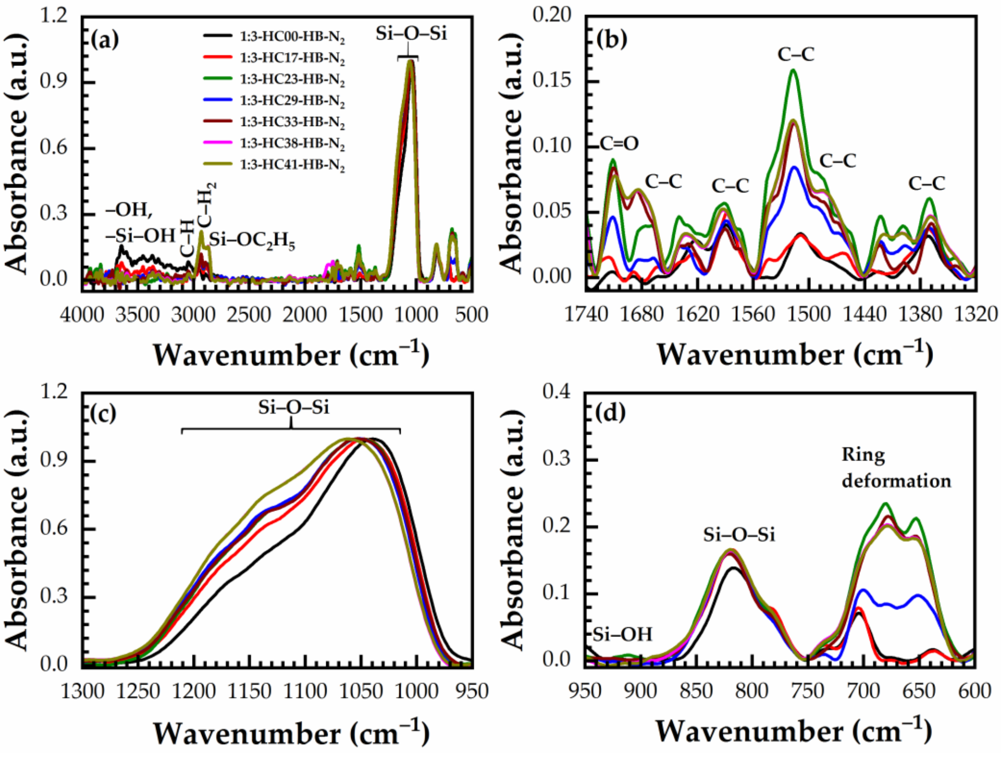

Figure S2. FTIR spectra of 1:3 ratio material films hard baked (HB) in N_2 (60 min) with different template loadings (0 to 41 wt%, films numbered from HC17 to HC41); where (**a**) full FTIR spectra; and (**b–d**) are the parts of the full FTIR spectra.

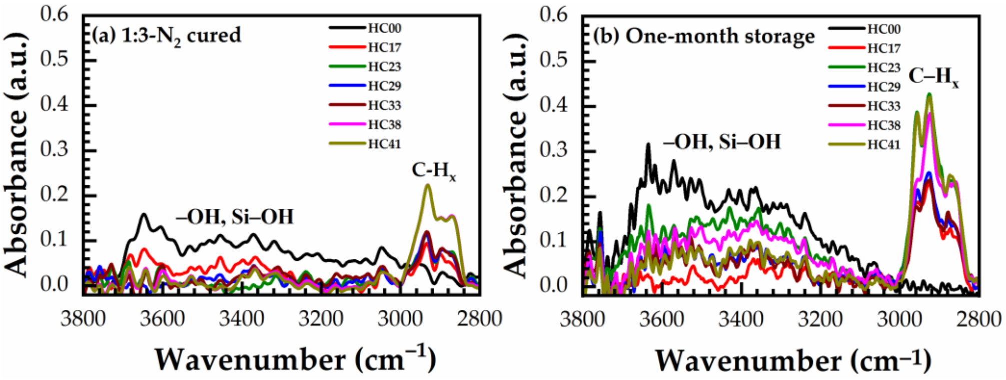

Figure S3. Fingerprint region (3800–2800 cm^{-1}) of FTIR spectra of (**a**) N_2 cured for 60 min (just cured) and the corresponding (**b**) one-month stored samples with different porogen (0 to 41 wt%, films numbered from HC17 to HC41) loadings.

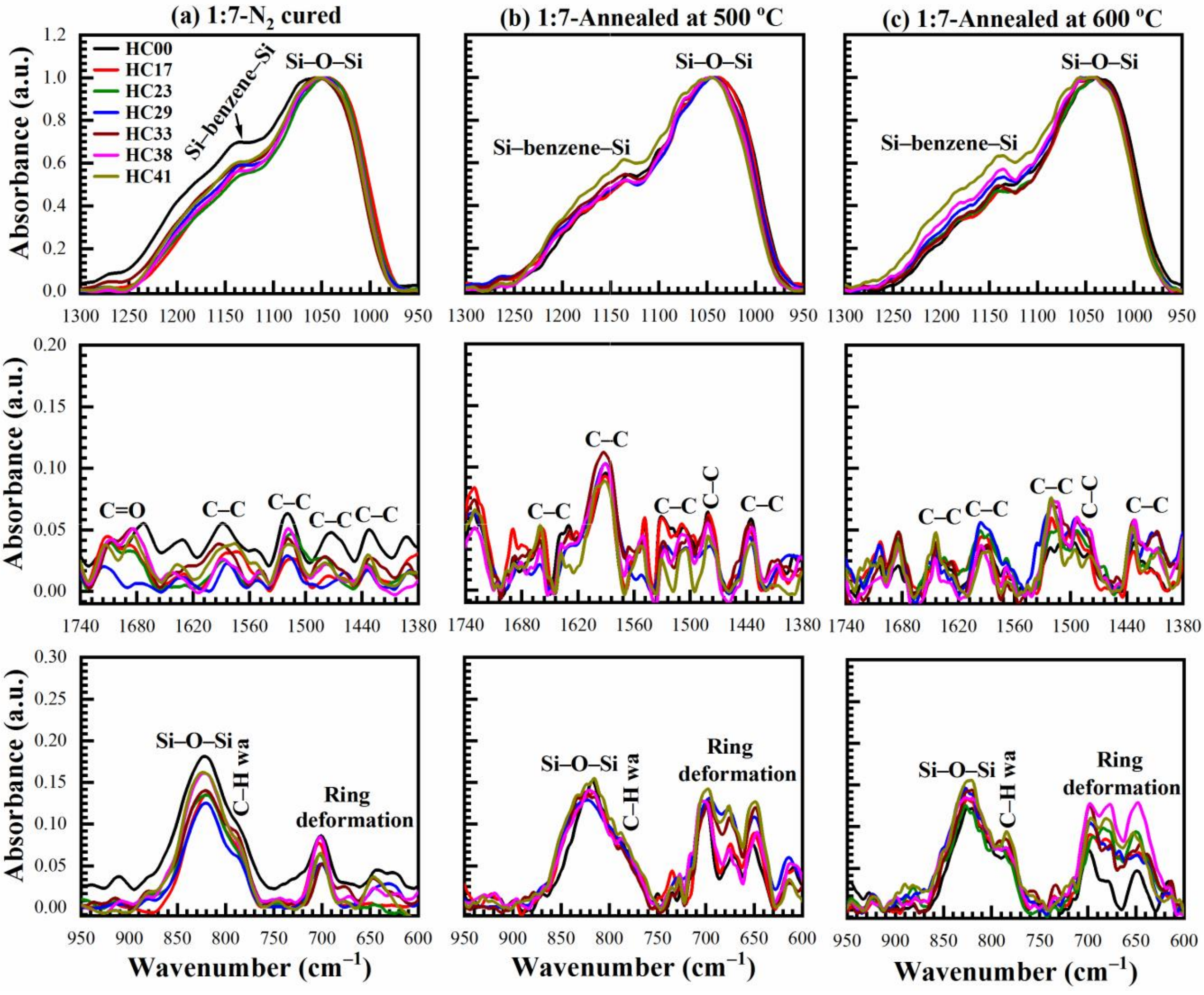

Figure S4. Ranges of FTIR spectra for 1:7 material after (**a**) hard baking (HB) at 400 °C in N_2 for 60 min (N_2 cured), (**b**) annealing at 500 °C for 30 min, and (**c**) annealing at 600 °C for 30 min with different porogen (from 0 to 41 wt%, films numbered from HC17 to HC41) loadings.
